# Diet Restriction Impact on High-Fat-Diet-Induced Obesity by Regulating Mitochondrial Cardiolipin Biosynthesis and Remodeling

**DOI:** 10.3390/molecules28114522

**Published:** 2023-06-02

**Authors:** Qiaoyu Li, Yuqi Lin, Jinlin Xu, Yukun Liu, Yuxuan Jing, Rongzeng Huang, Chengwu Song, Lijun Zhang, Shuna Jin

**Affiliations:** 1 College of Pharmacy, Hubei University of Chinese Medicine, 16 Huangjiahu West Road, Wuhan 430065, China; 2 College of Basic Medicine, Hubei University of Chinese Medicine, 16 Huangjiahu West Road, Wuhan 430065, China

**Keywords:** diet restriction, obesity, cardiolipin, targeted lipidomics, mitochondria

## Abstract

Diet restriction (DR) ameliorates obesity by regulating mitochondrial function. Cardiolipin (CL), a mitochondrial phospholipid, is closely associated with mitochondrial function. This study aimed to evaluate the anti-obesity effects of graded levels of DR based on mitochondrial CL levels in the liver. Obese mice were treated with 0%, 20%, 40%, and 60% reductions in the normal diet compared to normal animals (0 DR, 20 DR, 40 DR, and 60 DR groups, respectively). Biochemical and histopathological analyses were performed to evaluate the ameliorative effects of DR on obese mice. The altered profile of mitochondrial CL in the liver was explored using a targeted metabolomics strategy by ultra-high-pressure liquid chromatography MS/MS coupled with quadrupole time-of-flight mass spectrometry. Finally, gene expression associated with CL biosynthesis and remodeling was quantified. Tissue histopathology and biochemical index evaluations revealed significant improvements in the liver after DR, except for the 60 DR group. The variation in mitochondrial CL distribution and DR levels showed an inverted U-shape, and the CL content in the 40 DR group was the most upregulated. This result is consistent with the results of the target metabolomic analysis, which showed that 40 DR presented more variation. Furthermore, DR led to increased gene expression associated with CL biosynthesis and remodeling. This study provides new insights into the mitochondrial mechanisms underlying DR intervention in obesity.

## 1. Introduction

The prevalence of obesity has increased globally, reaching pandemic levels and imposing significant economic costs on healthcare systems over the last 50 years [1,2]. Obesity can increase the risk of premature death and medical conditions such as cardiovascular disease [3]. There are several methods used to improve obesity, including medication use, exercise, liposuction, and diet restriction (DR). However, all of these methods are invasive, expensive, and time-consuming, except for DR [4]. As a basic method to ameliorate obesity, DR encompasses a variety of regimens characterized by nutrient and/or energy restrictions, leading to changes at the organismal level [5]. Compared to other anti-obesity strategies, DR is a safer, more effective, and non-pharmacological intervention.

However, the ideal food intake for optimal health remains controversial. With the exception of a few existing studies, most of the available DR studies focused largely on a single level. Previous studies pointed out that 50% DR resulted in increased hydrogen sulfide production and protection from hepatic ischemia reperfusion injury, and 40% DR led to the browning of white adipose tissue through type 2 immune signaling [6,7]. In addition, the conclusions from different studies remain inconsistent. For example, as a common method of DR, calorie restriction is commonly prescribed to improve cardiac structure and function [8]; however, Yu confirmed that a 45% calorie restriction decreased cardiac function and decreased heart rate [9]. Meanwhile, DR at different levels diversely affects the physiological and psychological aspects of the organism, including body composition, behavioral phenotype, oxidative stress and basal metabolic rate [10,11,12]. Consequently, there is a lack of systemic evidence showing the appropriate food intake needed for health, and implementation of DR might require unique considerations to fill in the existing research gap.

Mitochondrial dysfunction contributes to oxidative stress and systemic inflammation, which are critical in obesity-related diseases [13]. In contrast, DR improves mitochondrial bioenergetics and dynamics by increasing efficiency, decreasing oxidant production, and increasing mitochondrial turnover [14,15,16]. However, mitochondrial mechanisms underlying DR remain unclear and require further investigation. Cardiolipin (CL), a mitochondria-specific phospholipid, is a valuable indicator of mitochondrial function in humans [17]. CL is localized in the mitochondrial inner membrane and exerts numerous biological functions, such as supporting the folding, sorting, and activity of respiratory chain components and regulating mitochondrial membrane dynamics [18,19]. Alterations in CL levels are strongly associated with mitochondrial function. Because the disruption of lipid metabolism could result in statistically significant changes in mitochondrial CL content, CL may be a critical regulator of mitochondrial health in such diseases [20,21]. Diabetes and obesity were characterized by CL deficiency and profound remodeling of CL’s acyl composition [22]. On the other hand, DR showed a significant increase in CL content measured with CL-dependent nonyl acridine orange staining signal [23]. The acyl length, oxidation, and saturation have different effects on the shape, binding, stability, and function of CL [24]. However, most previous studies have demonstrated the role of total CL changes in mitochondrial dysfunction or related diseases, and there remains a lack of evidence focusing on CL alone.

This study aimed to evaluate the anti-obesity effects of graded levels of DR based on mitochondrial CL levels in the liver. High-fat-diet-induced obese mice were treated with four levels of DR, including 0 DR, 20 DR, 40 DR, and 60 DR groups. Mitochondrial CL in the liver was identified and characterized by ultra-high-pressure liquid chromatography MS/MS coupled with quadrupole time-of-flight mass spectrometry (UHPLC-QTOF-MS/MS). Subsequently, the distribution of CL was compared based on the targeted lipidomic strategy in different levels of DR groups. The CLs with significant differences were screened to explore potential mitochondrial roles between DR and obesity. Finally, the expression of key genes involved in CL biosynthesis and remodeling was examined to search for their underlying mechanisms.

## 2. Results

### 2.1. Mitochondria Enrichment and Identification

**Figure 1:**
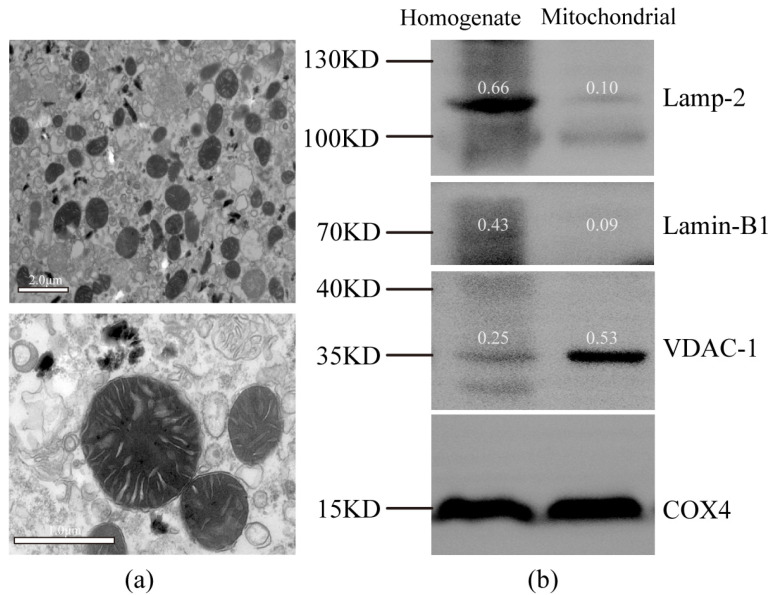
Purity evaluation of isolated mitochondria. (**a**) Transmission electron micrograph showing typical morphology of an isolated mitochondria-enriched fraction. (**b**) Western blot analysis of protein extracts from liver homogenate and an isolated mitochondria-enriched fraction (with COX4 as loading control). Lamp-2, lysosomal-associated membrane protein-2, lysosomal marker; Lamin-B1, nuclear marker; VDAC-1, voltage-dependent anion-selective channel-1, mitochondrial marker; COX4, cytochrome c oxidase-IV. The number above the belt represents the expression value relative to COX4.

The integrity of the mitochondrial fractions was examined by transmission electron microscopy. Intact inner and outer membranes were observed in the typical morphology of the isolated mitochondria, suggesting that the liver-enriched mitochondrial fractions were intact and sufficient for subsequent metabolic analysis (Figure 1a). The quality of mitochondrial separation was examined by Western blot using representative markers of different subcellular compartments, including lysosomes, nuclei, and mitochondria. The mitochondria-enriched fractions and tissue homogenates isolated from the liver were examined. The use of voltage-dependent anion-selective channel-1 (VDAC-1) as a mitochondrial marker is suitable because of its abundance on the mitochondrial outer membranes [25]. It was clear that lysosomal-associated membrane protein-2 (Lamp-2) and Lamin-B1 detected in the homogenate were more highly expressed than those in the enriched mitochondrial fraction; however, VDAC-1 was inversely expressed. (Figure 1b). This result further indicated that the method yielded good mitochondrial enrichment.

### 2.2. Physiological and Biochemical Parameters

**Figure 2:**
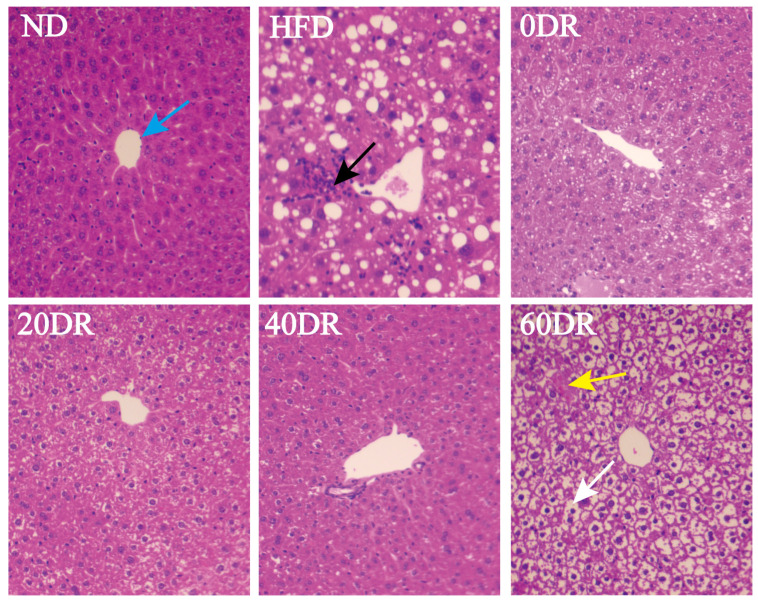
Microscopic findings in the liver tissue. Hematoxylin and eosin (HE) staining to visualize tissue morphology, central vein (blue arrow), inflammatory foci (black arrow), hepatic edema (white arrow), and focal hepatocyte necrosis (yellow arrow). Magnification = 100×.

Figure 2 shows the histological examination of the mouse liver using hematoxylin and eosin (HE) staining to explore the effect of DR on hepatic lipid accumulation. Hepatic cells in the normal diet (ND) group exhibited normal hepatocellular architecture with a normal central vein (blue arrow), and the structure of the liver lobule was clear. However, the livers of the high-fat diet (HFD) group showed obvious inflammatory foci (black arrow) and numerous large cytoplasmic lipid droplets (clear spaces). This phenomenon in the 0 DR, 20 DR, and 40 DR groups had improved to different degrees. In contrast, the liver of mice in the 60 DR group was observed with intracytoplasmic vacuoles with hepatic edema (white arrow) and focal hepatocyte necrosis (yellow arrow).

The physiological and biochemical characteristics of the experimental groups are presented in Table 1. Statistically significant differences in all indices were observed in the HFD group compared with the ND group (*p* < 0.05). Body weights in the four DR groups were significantly lower than those in the HFD group, and body weights in the 40 DR and 60 DR groups were also significantly lower than those in the ND group. A significant increase in body mass index (BMI) in the first stage of the HFD and all DR groups indicated the successful establishment of an HFD-induced obesity model. In the second stage, BMI was significantly reduced in all DR groups compared with that in the HFD group. Moreover, BMI was significantly more reduced in the 20 DR, 40 DR, and 60 DR groups than in the ND group. The liver weights were also decreased in all DR groups compared to the HFD group, and, similarly, liver weights were significantly more decreased in the 0 DR, 40 DR, and 60 DR groups than in the ND group. The concentrations of serum total cholesterol (TC), high-density lipoprotein cholesterol (HDL-C), and low-density lipoprotein cholesterol (LDL-C) showed an obvious decrease in all DR groups compared to those in the HFD group. There was no significant difference in TC levels between the 20 DR and 40 DR groups compared with the ND group. In addition, the HDL-C level in all DR groups was different from that in the ND group, and no significant difference in the LDL-C level was observed between all DR groups and the ND group.

### 2.3. Identification of Mitochondrial CLs in the Liver

The CLs share structural similarities (Figure S1). They mainly consist of four fatty acid chains, three glycerol groups, and two phospholipid groups. Because of their similar core structures, a variety of regular product ions were obtained. In the negative mode, [PA − H]^−^ (PA, phosphatidic acid), [M − R]^−^ (R, C13H27COO), and fatty acyl ions were the main characteristic fragment ions of the CLs. Before analyzing the CL species, the CL (18:1)_4_ standard was detected and characterized primarily as [M − H]^−^ ions in mouse liver mitochondria. The ions at *m*/*z* 591.4043, 1011.6308, and 227.2006 were the most sensitive diagnostic product ions for CL (18:1)_4_ (Figure S1).

**Figure 3:**
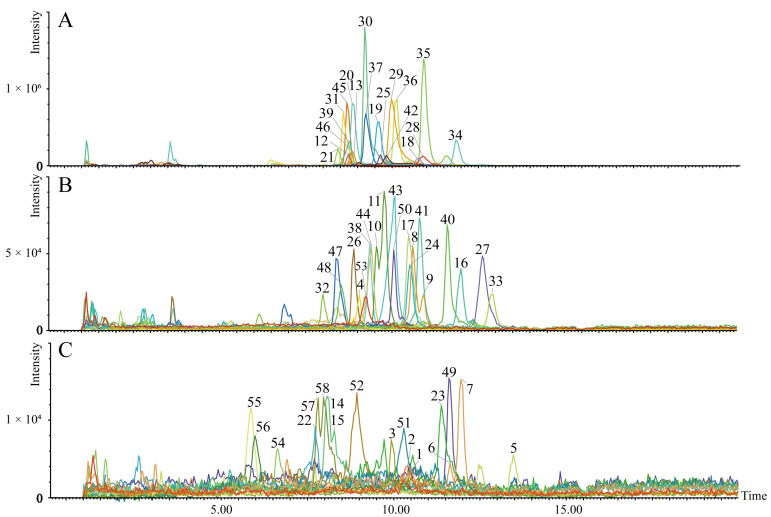
The established metabolite profile of 58 CLs in liver mitochondria. The extracted ion chromatograms (**A**–**C**) of the 58 targeted CLs ((**A**), CLs with peak intensity >1.01 × 10^5^ cps; (**B**), CLs with peak intensity >2.20 × 10^4^ and <1.01 × 10^5^ cps; (**C**), CLs with peak intensity >2.18 × 10^3^ and <2.00 × 10^4^ cps). The number on the peak represents the compound number.

Mitochondrial CL profiles in the liver were obtained by integrating the 58 CLs using UHPLC-QTOF-MS/MS (Figure 3). The UHPLC-QTOF-MS/MS information of the characterized mitochondrial CL in the liver is presented in Table S1.

### 2.4. Distribution of Mitochondrial CLs in the Liver

**Figure 4:**
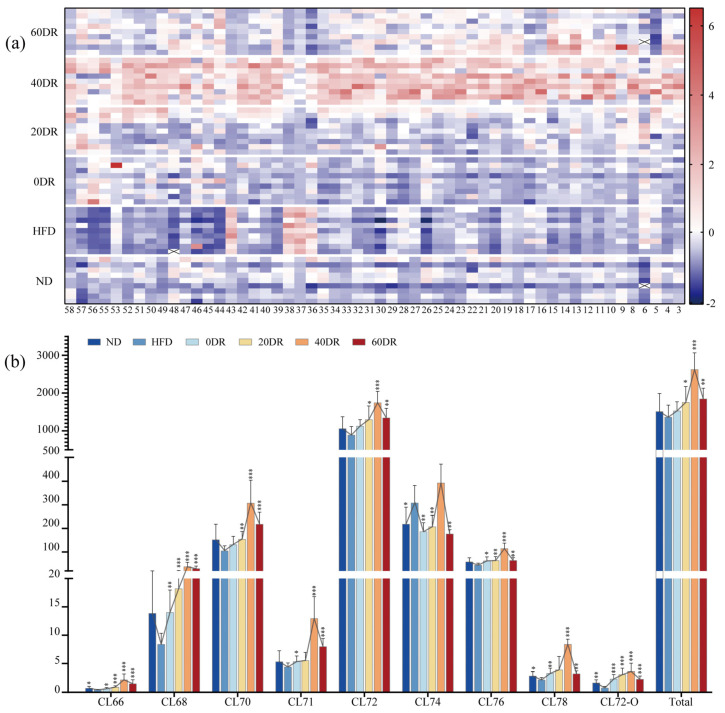
(**a**) The contents of 54 CLs detected in the experimental groups. The value of each compound was based on the z-score-normalized levels for better visualization. The color intensity from blue to red reflected the relative content of each CL. (**b**) Relative contents of total CL and nine CL species stratified by the number of C atoms in the liver mitochondria. The connected line of each metabolite indicates the changing trend. * *p* < 0.05, ** *p* < 0.01, *** *p* < 0.001 significantly different from HFD group.

In this study, 58 CL compounds were detected in the extracts of liver mitochondria, and there were 54 CLs for further analysis (the low abundance of the other four CLs led to inaccurate results). An overview of the distribution of 54 CLs in each sample is shown as a heatmap in Figure 4a. In the HFD group, most CLs are shown in a darker blue color, indicating a low CL level in the liver mitochondria in this group. There was a noticeable red color in the same CL in the 40 DR group, indicating that the 40 DR group had the most pronounced increase in CL content. In addition, the CL content in the other groups was between those of the above two groups.

To further analyze the data, the CLs were classified (Figure 4b). The column chart depicts the relative content of the total CL and each type of CL stratified by the number of C atoms, including CL66, CL68, CL70, CL71, CL72, CL74, CL76, CL78, and CL72-O. Among the nine CL species, the CL72 group was the most anteriorly distributed in the liver mitochondria, the content of which was dominated by the total CL content. The total CL content in the liver mitochondria among the six groups was in the following order: 40 DR > 60 DR > 20 DR > 0 DR > ND > HFD. Furthermore, the content of all individual CL species showed the same trend, except for the CL74 group, which showed an increase in the HFD group compared to that in the ND group.

### 2.5. Targeted Metabolomic Analysis of Mitochondrial CLs in the Liver

**Figure 5:**
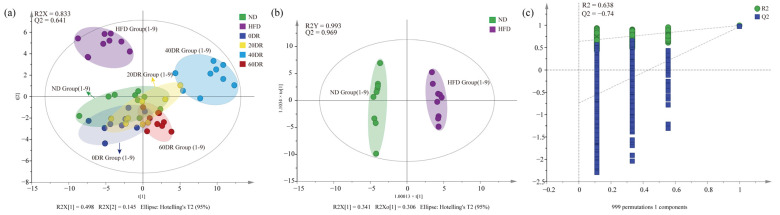
(**a**) The principal component analysis (PCA) score scatter plot of CL of six group samples. (**b**) The orthogonal partial least squares discriminant analysis (OPLS-DA) score scatter plots obtained from the ND and HFD groups. (**c**) The 999-permutation tests of OPLS-DA score scatter plots from the ND and HFD groups.

To further explore the regulatory role of CLs in the amelioration of obesity induced by graded DR, a targeted metabolomic strategy was used to screen for CLs in different groups. A scoring plot of principal component analysis (PCA) was performed to verify the repeatability of the present method and differentiation between groups (Figure 5). The separation was significant (R^2^X = 0.833, Q^2^ = 0.641), showing a difference in the content of CLs among the six groups. The results showed that the ND and HFD groups were completely separated and distant, and the difference remained after the graded DR.

The orthogonal partial least squares discriminant analysis (OPLS-DA) score plots for the ND group and HFD groups showed a clear separation (Figure 5b), and the results (R^2^Y = 0.993, Q^2^ = 0.969) indicated that the models had good abilities for both prediction and reliability. Variable importance in the projection (VIP) values was statistically estimated using the OPLS-DA model for different groups for subsequent analysis. A 999-permutation test was performed on the aforementioned model. The values of predicted R^2^ and Q^2^ from the regression lines were 0.638 and −0.74, respectively, which were both smaller than those from the actual models, indicating that the OPLS-DA model did not overfit (Figure 5c). The OPLS-DA models of other groups are shown in Figure S2.

### 2.6. The Screening of Mitochondrial CLs Related to DR Ameliorate Obesity

CLs that met the following three criteria were regarded as differential metabolites compared to the HFD group. First, if the VIP values in the corresponding OPLS-DA model were greater than 1.0. Second, if the CL content was significantly different (*p* < 0.05). Third, if the fold changes of CLs were >1.50 or <0.75. Consequently, a total of 23 CLs were selected in the ND group, including 15, 18, 19, and 18 differential CLs in the 0 DR, 20 DR, 40 DR, and 60 DR groups, respectively. However, when the fold changes were further confined (fold change > 2.0 and fold change < 0.5) to identify CLs with greater variability, there were 10, 10, 17, and 14 differential CLs in the 0 DR, 20 DR, 40 DR, and 60 DR groups, respectively. The VIP, *p*, and fold change values of the 23 CLs are presented in Table S2.

**Figure 6:**
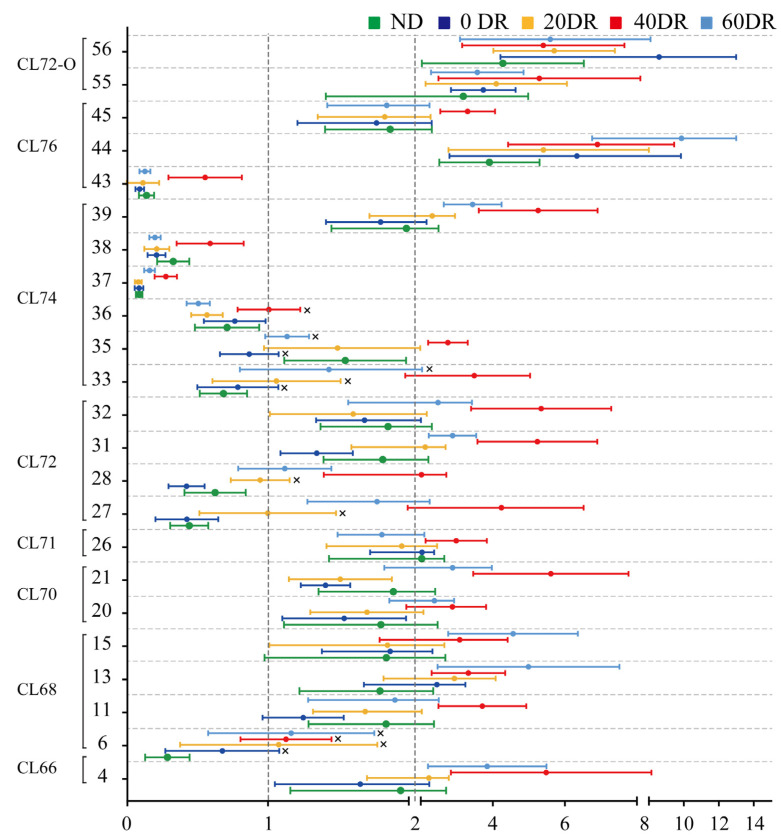
Changed multiples of the relative contents of 23 significant differential CLs detected in the other five groups compared to the HFD group. The abscissa is the fold change value and the ordinate is the compound number. × indicates non-significant differences (*p* > 0.05) compared with the HFD group.

For further quantitative analysis, the changes in the contents of the 23 CLs were analyzed (Figure 6). Each of the 23 CLs in the ND and DR groups was assessed in terms of their basic levels in the HFD group. The points on the right side of the abscissa = 1 indicate that the CL content increased in comparison with the HFD group, and the points on the left side represent the opposite. There was an overall upward trend in CL content after DR. There were several CLs whose contents were clearly reduced in both the ND and DR groups, such as compounds 6, 36, 37, 38, and 43, compared to the HFD group. Most of the differential CLs in the 40 DR group were at the far right of Figure 6, which indicates that 40 DR promoted a greater effect on mitochondrial CL growth than other DR levels.

### 2.7. The Biosynthesis and Remodeling Gene Expression of CLs

**Figure 7:**
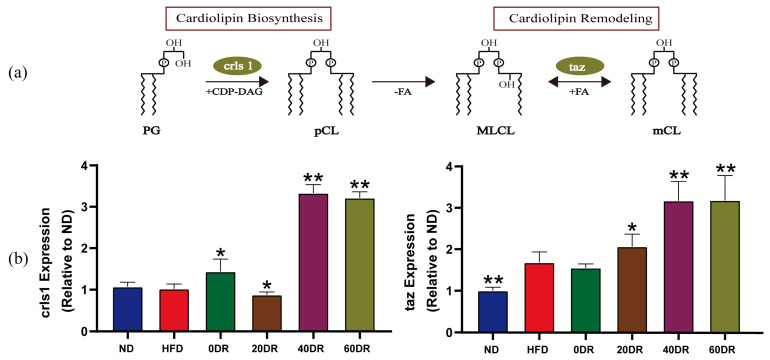
(**a**) Biosynthesis and remodeling of CL. PG: phosphatidylglycerol; crls1: cardiolipin synthase 1; CDP-DAG: cytidine diphosphate-diacylglycerol; pCL: premature CL; PLA: phospholipase A; FA: fatty acid; MLCL: monolysocardiolipin; taz: tafazzin; mCL: matured CL. (**b**) Different levels of DR to modulate CL biosynthesis and remodeling (*n* = 6). The expression of crls1 and taz. Mann–Whitney U test was used to calculate the significant difference. * *p* < 0.05, ** *p* < 0.01 significantly different from HFD group.

To further explore the basis of this graded response, we measured the messenger RNA expression, including cardiolipin synthase 1 (crls1) and tafazzin (taz). The crls1 is closely involved in the regulation of CL biosynthesis while taz is involved in the regulation of CL remodeling. CL biosynthesis and remodeling are key factors in the modulation of CL metabolic function. After de novo CLs synthesis and acyl remodeling, different CLs were prepared. First, CLs were formed by the condensation of one molecule of phosphatidylglycerol and one molecule of cytidine diphosphate-diacylglycerol by crls1. Subsequently, remodeling was performed using enzymes, such as taz, which converted the acyl chains of nascent CLs to mature CLs; this process was a bidirectional modulation (Figure 7a).

There was no significant difference in the expression of crls1 between the ND and HFD groups (Figure 7b). The expression of crls1 in the DR groups was significantly higher than that in the HFD group, except in the 20 DR group, where it was significantly lower than that in the HFD group. Taz expression was increased in the HFD group. The expression of taz was markedly increased in the 20 DR, 40 DR, and 60 DR groups compared to the HFD group, which showed no difference from the 0 DR group. In particular, higher expression of crls1 and taz was observed in both the 40 DR and 60 DR groups.

## 3. Discussion

In this study, we investigated the role of CL in the amelioration of obesity induced by DR to discover a theoretical basis for targeted treatment. Targeted metabolomic analysis based on UHPLC-QTOF-MS/MS was used to identify and distinguish variations in CL at different DR levels. Genes related to CL synthesis and remodeling were further quantified to explore their underlying mechanisms.

CL plays a pleiotropic role in regulating mitochondrial bioenergetic processes and inner membrane stability, including mitochondrial lamellar cristae formation, respiratory chain complexes, mitochondrial substrate carriers, association of enzymes with the inner mitochondrial membrane, and ATP synthesis [26]. In the progress of obesity and type 2 diabetes, CL deficiency plays a crucial role in mitochondrial dysfunction [22]. A previous study demonstrated that liver mitochondrial dysfunction occurs when CL and/or tetralinoleoylcardiolipin content was reduced by 35% [27]. Rats with non-alcoholic fatty liver disease have decreased levels of CL but increased levels of peroxidized CL in the liver tissue [28].

Our data indicated an absolute decrease in mitochondrial CL concentration in the liver of obese mice, which is in line with the results of other studies [29,30]. This reduction was associated with CL damage, which contributed to an increase in electron leakage from the electron transport chain, generation of more superoxide radicals, and perpetuation of a cycle of oxygen-radical-induced mitochondrial membrane damage, all of which ultimately led to liver damage. During this process, mitochondrial lipid peroxidation is induced, which generally occurs in organs such as the heart, liver, placenta, kidneys, and subcutaneous adipose tissue in individuals with diabetes and obesity [31].

Our data confirmed that DR induced a considerable increase in CL concentrations in the liver mitochondria of obese mice. This result demonstrates that DR is of particular interest for liver mitochondrial membrane lipids. Here, DR alters the physical properties of mitochondrial membranes by increasing CL density. DR might be a vital promoter of energy factors in the liver tissue by upregulating CLs to diminish energy-metabolism-associated disturbances. In addition, calorie restriction can promote CL distribution between mitochondrial membranes due to the additional CL caused by calorie restriction in the mitochondrial outer membranes [32]. On this basis, several phenomena can be generated by DR, such as a decrease in oxygen consumption, membrane potential, and reactive oxygen species, to induce mitochondrial biogenesis and bioenergetic efficiency [23]. Through a series of comprehensive analyses, some differential CLs indicated different variations between obese mice and mice with different levels of DR. Detailed information on mitochondrial CL may explain the unique efficacy of DR, a basic method to ameliorate obesity. These different CLs could contribute to the mitochondrial mechanism of action or play a role in DR and obesity.

Maintaining normal species and CL content is crucial for mitochondrial function and structural integrity. Our study pinpointed the difference in CL content after graded levels of DR. The level of DR did not have a proportional effect on CL content, and the associations had an inverted U-shape. Our results showed that 40% DR had a more dramatic effect on mitochondrial CL regulation in the liver than 0%, 20%, or 60% DR in obese mice. A previous study indicated that calorie restriction could promote the biosynthesis and remodeling of the CL [32]. This was consistent with our results that the expression of crls1 and taz was higher after 40% and 60% DR. From the DR intervention on obesity, the 40 DR group could repair severe hepatocyte steatosis, but the liver of 60 DR mice showed marked striking intracellular edema and focal hepatocellular necrosis. Based on these results, we provide evidence that 40% DR achieved relatively good amelioration of obesity.

## 4. Materials and Methods

### 4.1. Chemicals and Materials

Commercial kits for TC, HDL-C, and LDL-C were purchased from Jiancheng Bio (Nanjing, China). Radioimmunoprecipitation assay (RIPA) lysis buffer was purchased from ServiceBio (Wuhan, China). HPLC-grade methanol, chloroform, and acetonitrile were obtained from Fisher Scientific (Waltham, MA, USA). Antibodies against cytochrome c oxidase IV (COX4), VDAC-1, Lamp-2, and Lamin-B1 were obtained from ServiceBio (Wuhan, China). Sodium dodecyl sulfate, 2.5% glutaraldehyde, polyacrylamide gel electrophoresis, Tween-20 (TBST), phosphate buffer (PB, pH 7.4), and osmic acid were purchased from ServiceBio (Wuhan, China). TRIzol reagent was obtained from Bioleuki (Wuhan, China). The primers were purchased from Sangon (Shanghai, China).

### 4.2. Animals and DR

The Institutional Animal Care and Use Committee of Hubei University of Chinese Medicine approved all animal experiments. The Huazhong University of Science and Technology (Wuhan, China) provided 54 Kunming mice (20–25 g). Laboratory animal certificate number: SCXK (e) 2017-0067. All experimental animals were individually housed in cages with standard conditions (temperature, 23 ± 2 °C; humidity, 55% ± 5%; 12 h light/dark). In this animal experiment, normal and high-fat diets were prepared. The HFD formula was as follows: 78.8% normal feed, 10% lard, 10% egg yolk, 1% cholesterol, and 0.2% cholate [33]. All mice were fed for one week for adaptation and then randomized into six groups (nine mice each): the normal mice + ND (ND), obese mice + HFD (HFD), obese mice + normal diet (0 DR), obese mice + 20% normal diet DR (20 DR), obese mice + 40% normal diet DR (40 DR), and obese mice + 60% normal diet DR (60 DR) groups. All groups were treated with a two-step dietary plan. In the first stage, the ND group was fed a normal diet, and all other groups were fed a high-fat diet. Two weeks of the high-fat diet resulted in mice with a BMI ≥ 310, which was considered obese [34]. The BMI of the mice was evaluated using the Lee index [35]. In the second stage, the diet of the mice in the ND and HFD groups remained unchanged, the 0 DR group was replaced with a normal diet ad libitum, and the 20 DR, 40 DR, and 60 DR groups were also replaced with a normal diet but reduced by 20%, 40%, and 60% relative to the ND group, respectively. The second stage lasted for 2 weeks.

### 4.3. HE Staining and Morphometric Analysis

To examine the effects of DR on HFD-induced hepatic steatosis, tissue samples were collected for histological examination immediately after the mice were anesthetized and sacrificed by cervical dislocation at the end of the study. Briefly, fixed liver specimens were paraffin-embedded, sectioned at 4 μm, and subjected to HE staining for evaluating the pathologic changes. Images from six different groups of sections were analyzed using Imageview software version 3.7.

### 4.4. Biochemical Analysis

The experimental period for the entire dietary intervention was 28 days. Food consumption was monitored daily, and body weight and nasal–anal length were measured every 3 days. After completion of the second stage, the fasting whole blood samples were obtained by ophthalmectomy under anesthesia (isomobarbital, 100 mg·kg^−1^) and finally collected into clean test tubes without anticoagulant. Serum samples were separated by centrifugation. Serum TC, HDL-C, and LDL-C concentrations were measured using an enzymatic colorimetric method and the values were determined using an Infinite F50 microplate reader (Tecan, Grödig, Austria).

### 4.5. Isolation and Purity Verification of Mitochondria

#### 4.5.1. Isolation of Mitochondria

Differential centrifugation was used to enrich the mitochondria in the mouse liver using a mitochondrial isolation kit purchased from Biovision (Milpitas Blvd, Milpitas, CA, USA) [36]. Briefly, after the livers were collected, washed, and homogenized using pre-cooled glass homogenizers, the resulting homogenates were immediately centrifuged at 600× *g* for 10 min with the isolation buffer provided by the kit. Subsequently, the supernatant was carefully collected and centrifuged at 7000× *g* for 10 min to precipitate the mitochondria, which were washed again by centrifugation with an isolation buffer. Finally, the supernatants were removed and the mitochondria were re-suspended in the storage buffer provided with the kit.

#### 4.5.2. Western Blot Analysis

Proteins from purified mitochondria and liver homogenate were extracted with RIPA lysis buffer, resolved by 10% SDS-PAGE at 120 V, transferred to 0.45 mm PVDF (polyvinylidene fluoride) for 30 min at 300 mA, and finally analyzed by immunoblotting. All membranes were blocked with 5% non-fat dry milk in Tris-buffered saline containing Tween 20 (TBST) for 30 min at 23 ± 2 °C following incubation with primary antibodies in milk at 4 °C. At the specified dilutions, primary antibodies were used against the following proteins: Lamp-2 (120 KD, 1:1000), Lamin-B1 (69 KD, 1:1000), and VDAC-1 (32 KD, 1:5000). After washing with TBST thrice for 5 min each, the membranes were incubated with the appropriate secondary antibodies in milk (1/5000) for 30 min at room temperature. Finally, the membranes were washed three times for 5 min each with TBST. Visualization was performed by enhanced chemiluminescence staining.

#### 4.5.3. Transmission Electron Microscopy

Enriched mitochondria were immersed in a solution of glutaraldehyde solution (2.5%) for 3 h at 4 °C, washed in 0.1 M PB (pH = 7.4), and postfixed for 2 h in the dark, at room temperature, with 1% osmic acid. The samples were subsequently washed three times in phosphate buffer, dehydrated using a graded series of 50%–100% ethanol solutions and 100% acetone, and embedded in paraffin. Sections at 812.70 nm were counterstained for 15 min with 2% uranyl acetate in ethanol and lead citrate. This was followed by observation under an HT7700 transmission electron microscope (Hitachi High-Tech Co., Tokyo, Japan).

### 4.6. Extraction of CL from Isolated Liver Mitochondria

CL extraction was completed with minor adjustments, as described in previous reports [37]. Briefly, purified mitochondria were added sequentially to 3 mL of a CHCl_3_/MeOH 1/1 (*v*/*v*) mixture and 1.8 mL of 9% NaCl, vortexed for 30 s, and ultrasonically extracted for 10 min. The resulting mixture was centrifuged at 1425× *g* for 10 min at room temperature (23 °C). After the phase separation, the bottom layer (chloroform layer) was collected. The supernatant was recovered and a second extraction was performed using 2.0 mL of chloroform. The combined extracts were aspirated, dried by blowing with N2, and reconstituted in 200 μL 1/1 (*v*/*v*) acetonitrile/isopropanol. Finally, the supernatant was centrifuged at 12,830× *g* for 10 min and collected in sample vials. Standard CL (14:0)_4_ with a final concentration of 100 ng mL^−1^ was used not only as an internal standard but also as an inter-batch quality control (QC) sample.

### 4.7. Identification of Mitochondrial CLs by UHPLC-QTOF-MS/MS

Reversed-phase liquid chromatography was performed on the C18 column (100 mm × 2.1 mm i.d., 1.7 µm, Waters, Milford, MA, USA) with 5 mM ammonium formate water (A) and 5 mM ammonium formate in methanol/2-propanol (1:1, *v*/*v*) (B). The UHPLC column was maintained at 40 °C with a flow rate of 0.3 mL/min and the injected volume of each sample was 2.0 μL. The LC gradient elution conditions followed a binary gradient with linear interpolation: 0 min, 90% B; 6 min, 95% B; 15 min, 98% B; 18 min, 98% B; 18.1 min, 90% B; and 20 min, 90% B. Samples were analyzed on a Waters Xevo G2-XS QTof mass spectrometer equipped with an electrospray ionization source. The sequential negative ion method of MS data collection was carried out with the following MS tuning parameters: capillary voltage 2.5 kV, cone gas flow 50 L·h^−1^, source temperature 100 °C, desolvation temperature 500 °C, desolvation gas flow 500 L·h^−1^, cone voltage 40 V. The MS data were acquired in the MSE continuum mode, and the full scans ranged from 50 to 1800 Da with a scan duration of 1 s.

CLs were matched based on the retention time and charge–mass ratio according to a previously established method [38]. The chromatographic behavior of the products and precursor ions was examined for presence and consistency. Correspondingly, unreasonable ions such as isotopic and false-positive peaks were excluded during the matching process.

### 4.8. Targeted Metabolomic Analysis

Targeted metabolomic analysis was performed on mitochondrial CLs in the livers of all groups. Following mitochondrial CLs identification, CLs were quantified based on their relative abundance (the relative area of the corresponding peak). The relative content of each CL was calculated as the CL peak area divided by the CL peak area (14:0)_4_. Multivariate statistical analysis was based on the transformed relative quantitation results (log10 transformation), and missing values were set at 1 × 10^−9^. Multivariate statistical analysis with unit variance (UV) scaling methods was performed on the entire data table during target metabolomic profiling.

To explore the role of DR-induced CL compounds caused by DR in the amelioration of obesity, PCA was used for multivariate exploration of clusters and trends among the six groups. Subsequently, OPLS-DA was used to identify clusters and trends in the ND and HFD groups. Moreover, 999 random-permutation tests were performed to investigate the overfitting of the OPLS-DA model, and R^2^Y and Q^2^ were calculated for goodness-of-fit and goodness-of-prediction, respectively. VIP values were calculated using the OPLS-DA model. The importance of the variables for classification was measured based on whether the VIP value was greater than 1.0 [39,40]. Significantly differential CLs were further screened according to their VIP values.

### 4.9. Real-Time Polymerase Chain Reaction (RT-PCR) Analysis

Total RNA was isolated from the liver using TRIzol and quality-checked using 260/230 nm and 260/280 nm scores added to a NanoDrop 2000/2000C micro nucleic acid protein concentration analyzer (Thermo Fisher Scientific, Waltham, MA, USA). The SweScript RT II First Strand cDNA Synthesis Kit (Servicebio, Wuhan, China) was used to reverse transcribe the mRNAs into cDNAs. The cDNAs were subsequently mixed with the indicated primers and 2 × Universal BlueSYBR Green qPCR Master Mix (Servicebio, Wuhan, China) for PCR detection. RT-PCR was performed using the CFX96 Real-Time PCR System (Bio-Rad, , Hercules, CA, USA). The following oligonucleotides were used: crls1 forward ATCCTTGCTATGCCACTGCT and reverse AAACTGGAGCTGCCAGAGAA; taz forward GAATTGGACGGCTGATTGCT and reverse GGAAGTAGGGTGGGCTGTTA.

### 4.10. Data Analysis

MassLynx 4.1 software (Waters, Milford, MA, USA) was utilized not only to operate and process the UHPLC-QTOF-MS/MS system but also to process and analyze the MS/MS spectra. Statistical analysis was performed using multivariate analysis with SIMCA-P (v14.1, Umetrics, Umeå, Sweden). Significant differences were analyzed with a Mann-Whitney U test using SPSS (version 26.0; IBM, Armonk, NY, USA). Statistical significance was defined as *p* values < 0.05. GraphPad Prism 8.0.1 (GraphPad Software Inc., San Diego, CA, USA) was used for visual analysis.

## 5. Conclusions

Our results showed that the mitochondrial CL content in the liver increased the most in the 40 DR group. In addition, the differential CLs caused by different levels of DR, based on the statistical model analysis, could serve as efficient selection indices for mitochondrial dysfunction in obesity. This has great practical significance in providing guidance for improving obesity based on mitochondrial mechanisms. Future studies will be dedicated to obtaining more transcriptomic response data on CLs changes after different degrees of DR for comprehensive analyses of mitochondrial mechanisms.

## Figures and Tables

**Table 1:** Physiological and biochemical parameters of mice (mean ± SD, *n* = 9).

	ND	HFD	0 DR	20 DR	40 DR	60 DR
Body weight (g)	42.86 ± 4.49 **	50.63 ± 3.06	46.24 ± 2.46 **	38.30 ± 4.15 ***	31.26 ± 3.43 ***^,#^	29.16 ± 0.95 ***^,###^
BMI at the first stage	309.15 ± 3.60 **	315.41 ± 2.00	316.58 ± 3.36 ^##^	316.16 ± 3.56 ^##^	316.65 ± 3.39 ^##^	316.60 ± 2.88 ^##^
BMI at the second stage	313.73 ± 6.86 **	330.18 ± 4.66	314.65 ± 9.41 ***	299.91 ± 7.82 ***^,##^	288.66 ± 5.99 ***^,###^	294.04 ± 3.96 ***^,###^
Liver weight	1.94 ± 0.23 *	2.27 ± 0.26	1.77 ± 0.14 ***^,#^	1.85 ± 0.29 **	1.47 ± 0.16 ***^,##^	1.42 ± 0.24 ***^,##^
TC (mmol L^−1^)	2.65 ± 0.29 ***	6.74 ± 0.91	3.23 ± 0.27 ***^,##^	2.54 ± 0.45 ***	2.88 ± 0.79 ***	3.22 ± 0.47 ***^,#^
HDL-C (mmol L^−1^)	2.05 ± 0.27 ***	3.20 ± 0.44	2.45 ± 0.33 **^,#^	1.61 ± 0.35 ***^,#^	1.65 ± 0.37 ***^,#^	1.63 ± 0.21 ***^,##^
LDL-C (mmol L^−1^)	0.45 ± 0.13 ***	1.26 ± 0.27	0.49 ± 0.08 ***	0.34 ± 0.12 ***	0.38 ± 0.18 ***	0.47 ± 0.13 ***

The results were presented as the means ± SD. The Lee index was calculated as the body mass index (BMI) of mice according to the following formula: [^3^√body weight (g)/length (mm)] × 10^4^; TC: total cholesterol, HDL-C: high-density lipoprotein cholesterol, LDL-C: low-density lipoprotein cholesterol. Mann–Whitney U test was used to calculate significant differences. * *p* < 0.05, ** *p* < 0.01, *** *p* < 0.001 significantly different from HFD group; ^#^ *p* < 0.05, ^##^ *p* < 0.01, ^###^ *p* < 0.001 significantly different from ND group.

## Data Availability

The datasets generated and/or analyzed during the current study are available from the corresponding author upon reasonable request.

## Supplementary Materials

The following supporting information can be downloaded at: https://www.mdpi.com/article/10.3390/molecules28114522/s1, Figure S1: The chemical structure and characteristic product ions of CL (18:1)_4_; Figure S2: OPLS-DA scores scatter plot of 0 DR, 20 DR, 40 DR, and 60 DR group vs. HFD group, respectively; Table S1: The detailed information of CLs obtained from liver mitochondria using UHPLC-QTOF-MS/MS; Table S2: The screened differential CLs in experimental groups.

## Sample Availability

Samples of the experimental samples are available from the authors.
