# Supplementary material for: Diet Restriction Impact on High-Fat-Diet-Induced Obesity by Regulating Mitochondrial Cardiolipin Biosynthesis and Remodeling
Source: Molecules. 2023 Jun 2;28(11):4522. doi: 10.3390/molecules28114522 (PMC10254315; doi:10.3390/molecules28114522)
Supplement: Supplementary file 1 [file molecules-28-04522-s001.zip › molecules-2294096-supplementary.pdf]

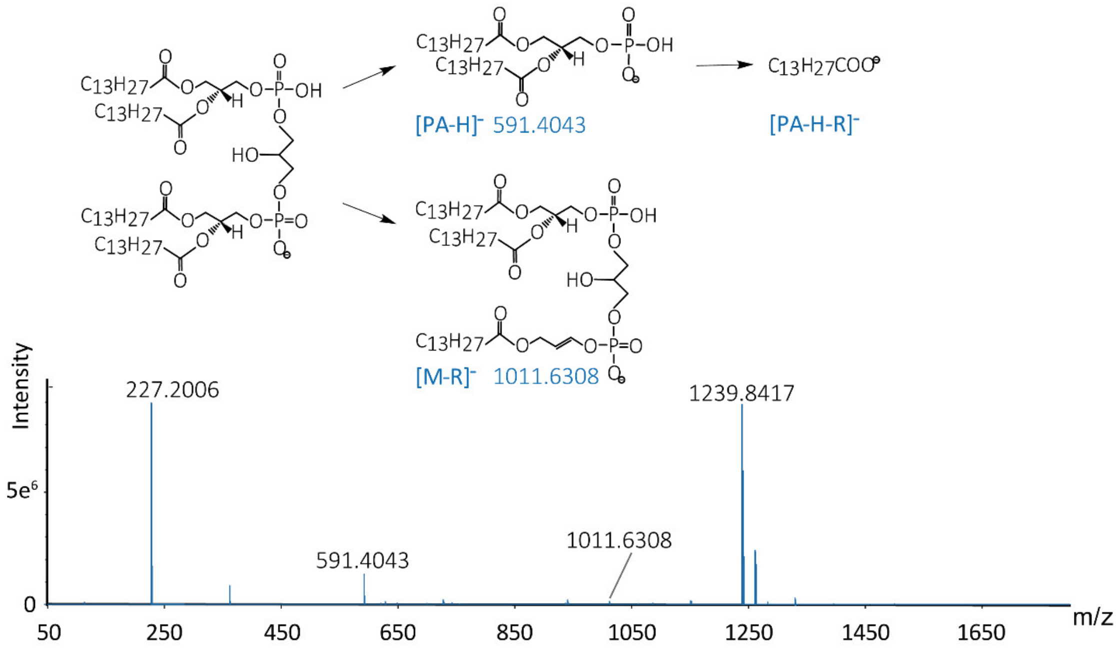

Figure S1. The chemical structure and characteristic product ions of CL (18:1)$_4$. PA: phosphatidic acid; R: $C_{13}H_{27}COO$.

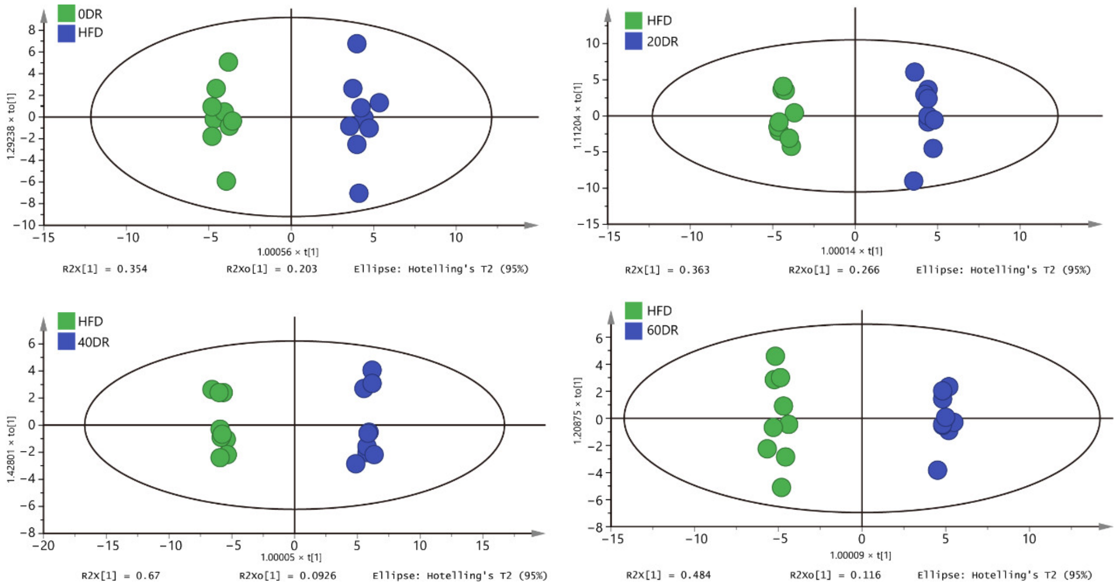

Figure S2. OPLS-DA scores scatter plot of 0 DR, 20 DR, 40 DR, and 60 DR group vs. HFD group, respectively. Group information: ND, normal diet group; HFD, high-fat diet group; 0 DR, 20 DR, 40 DR and 60 DR refer to 0%, 20%, 40% and 60% dietary restriction relative to the normal diet group, respectively.

Table S1. The detailed information of CLs obtained from liver mitochondria using UHPLC-QTOF-MS/MS.

No.	Molecular species	Formula	[M-H]-/exact	[M-H]-/measure	Mass error (PPM)	R1+R2	R3+R4	RT (min)
1	CL66:3	$C_{75}H_{140}O_{17}P_2$	1373.9488	1373.9480	0.58	643.4428	645.4625	10.25
2	CL66:3	$C_{75}H_{140}O_{17}P_2$	1373.9488	1373.9556	-4.95	/	/	10.39
3	CL66:4	$C_{75}H_{138}O_{17}P_2$	1371.9331	1371.9373	-3.06	643.4428	/	9.93
4	CL66:5	$C_{75}H_{136}O_{17}P_2$	1369.9175	1369.9204	-2.12	669.4493	643.4428	9.05
5	CL68:2	$C_{77}H_{146}O_{17}P_2$	1403.9957	1404.0092	-9.62	673.4795	/	13.46
6	CL68:3	$C_{77}H_{144}O_{17}P_2$	1401.9801	1401.9843	-3.00	699.4997	645.4625	11.64
7	CL68:3	$C_{77}H_{144}O_{17}P_2$	1401.9801	1401.9919	-8.42	671.4670	673.4795	11.95
8	CL68:4	$C_{77}H_{142}O_{17}P_2$	1399.9644	1399.9685	-2.93	697.4803	645.4486	10.60
9	CL68:4	$C_{77}H_{142}O_{17}P_2$	1399.9644	1399.9608	2.57	671.4692	/	10.88
10	CL68:5	$C_{77}H_{140}O_{17}P_2$	1397.9488	1397.9618	-9.30	695.4700	645.4486	9.55
11	CL68:5	$C_{77}H_{140}O_{17}P_2$	1397.9488	1397.9464	1.72	669.4493	671.4639	9.77
12	CL68:6	$C_{77}H_{138}O_{17}P_2$	1395.9331	1395.9336	-0.36	669.4493	/	8.74
13	CL68:6	$C_{77}H_{138}O_{17}P_2$	1395.9331	1395.9336	-0.36	693.4530	645.4521	8.88
14	CL68:7	$C_{77}H_{136}O_{17}P_2$	1393.9180	1393.9221	-2.94	695.4646	641.4232	8.07
15	CL68:7	$C_{77}H_{136}O_{17}P_2$	1393.9180	1393.9221	-2.94	667.4429	669.4546	8.28
16	CL70:4	$C_{79}H_{146}O_{17}P_2$	1427.9957	1428.0045	-6.16	699.4934	671.4724	12.00
17	CL70:5	$C_{79}H_{144}O_{17}P_2$	1425.9839	1425.9933	-6.59	697.4812	671.4692	10.48
18	CL70:5	$C_{79}H_{144}O_{17}P_2$	1425.9839	1425.9778	4.28	695.4656	673.4763	10.76
19	CL70:6	$C_{79}H_{142}O_{17}P_2$	1423.9644	1423.9757	-7.94	697.4812	669.4546	9.62

20	CL70:7	$C_{79}H_{140}O_{17}P_2$	1421.9488	1421.9519	-2.18	669.4493	695.4656	8.84
21	CL70:8	$C_{79}H_{138}O_{17}P_2$	1419.9331	1419.9294	2.61	693.4518	669.4470	8.43
22	CL70:9	$C_{79}H_{136}O_{17}P_2$	1417.9180	1417.9086	6.63	691.4313	669.4493	7.72
23	CL71:5	$C_{80}H_{146}O_{17}P_2$	1439.9957	1439.9989	-2.22	/	/	11.38
24	CL71:6	$C_{80}H_{144}O_{17}P_2$	1437.9801	1437.9789	0.83	/	/	10.52
25	CL71:7	$C_{80}H_{142}O_{17}P_2$	1435.9644	1435.9685	-2.86	/	/	9.67
26	CL71:8	$C_{80}H_{140}O_{17}P_2$	1433.9488	1433.9458	2.09	/	/	8.94
27	CL72:5	$C_{81}H_{148}O_{17}P_2$	1454.0114	1454.0142	-1.93	697.4812	699.4997	12.62
28	CL72:6	$C_{81}H_{146}O_{17}P_2$	1451.9957	1452.0081	-8.54	697.4812	/	10.91
29	CL72:7	$C_{81}H_{144}O_{17}P_2$	1449.9801	1449.9878	-5.31	695.4656	697.4812	9.98
30	CL72:8	$C_{81}H_{142}O_{17}P_2$	1447.9644	1447.9624	1.38	695.4656	751.4932	9.19
31	CL72:9	$C_{81}H_{140}O_{17}P_2$	1445.9488	1445.9436	3.60	693.4530	695.4656	8.59
32	CL72:10	$C_{81}H_{138}O_{17}P_2$	1443.9331	1443.9276	3.81	691.4325	695.4656	8.00
33	CL74:6	$C_{83}H_{150}O_{17}P_2$	1480.0270	1480.0300	-2.03	725.5083	697.4857	12.88
34	CL74:7	$C_{83}H_{148}O_{17}P_2$	1478.0114	1478.0061	3.59	723.5027	697.4886	11.84
35	CL74:8	$C_{83}H_{146}O_{17}P_2$	1475.9957	1475.9932	1.69	723.4973	695.4656	10.94
36	CL74:9	$C_{83}H_{144}O_{17}P_2$	1473.9801	1473.9780	1.42	721.4829	695.4656	10.14
37	CL74:10	$C_{83}H_{142}O_{17}P_2$	1471.9644	1471.9583	4.14	745.4881	669.4440	9.21
38	CL74:10	$C_{83}H_{142}O_{17}P_2$	1471.9644	1471.9583	4.14	719.4659	695.4656	9.32
39	CL74:11	$C_{83}H_{140}O_{17}P_2$	1469.9488	1469.9477	0.75	743.4670	669.4575	8.78
40	CL76:8	$C_{85}H_{150}O_{17}P_2$	1504.0270	1504.0283	-0.86	723.4918	/	11.62
41	CL76:9	$C_{85}H_{148}O_{17}P_2$	1502.0114	1502.0110	0.27	749.5187	695.4656	10.79

42	CL76:10	$C_{85}H_{146}O_{17}P_2$	1499.9957	1499.9937	1.33	721.4829	/	9.83
43	CL76:10	$C_{85}H_{146}O_{17}P_2$	1499.9957	1499.9937	1.33	747.4908	695.4656	10.08
44	CL76:11	$C_{85}H_{144}O_{17}P_2$	1497.9801	1497.9832	-2.07	801.5083	695.4646	9.36
45	CL76:12	$C_{85}H_{142}O_{17}P_2$	1495.9644	1495.9654	-0.67	743.4656	695.4656	8.69
46	CL76:12	$C_{85}H_{142}O_{17}P_2$	1495.9644	1495.9654	-0.67	719.4659	719.4659	8.85
47	CL76:13	$C_{85}H_{140}O_{17}P_2$	1493.9488	1493.9456	2.14	743.4670	693.4465	8.38
48	CL76:13	$C_{85}H_{140}O_{17}P_2$	1493.9488	1493.9456	2.14	719.4660	717.4517	8.52
49	CL78:11	$C_{87}H_{148}O_{17}P_2$	1526.0114	1526.0189	-4.91	749.5203	747.4924	11.6
50	CL78:12	$C_{87}H_{146}O_{17}P_2$	1523.9957	1524.0037	-5.25	719.4659	747.4908	10.05
51	CL78:12	$C_{87}H_{146}O_{17}P_2$	1523.9957	1524.0037	-5.25	801.5083	719.4659	10.29
52	CL78:13	$C_{87}H_{144}O_{17}P_2$	1521.9801	1521.9739	4.07	769.4884	695.4656	8.93
53	CL78:13	$C_{87}H_{144}O_{17}P_2$	1521.9801	1521.9739	4.07	747.4924	717.4517	9.24
54	CL72:8-O	$C_{81}H_{142}O_{18}P_2$	1463.9593	1463.9581	0.82	/	/	6.60
55	CL72:8-2O	$C_{81}H_{142}O_{19}P_2$	1479.9542	1479.9541	0.07	/	/	5.86
56	CL72:8-2O	$C_{81}H_{142}O_{19}P_2$	1479.9542	1479.9541	0.07	/	/	5.98
57	CL72:8-3O	$C_{81}H_{142}O_{20}P_2$	1495.9491	1495.9469	1.47	/	/	7.81
58	CL72:8-3O	$C_{81}H_{142}O_{20}P_2$	1495.9491	1495.9469	1.47	/	/	7.97

Table S2: The screened differential CLs in experimental groups.

	ND			0 DR			20 DR			40 DR			60 DR		
No.	VIP [a]	fold-change [b]	P-Value [c]	VIP [a]	fold-change [b]	P-Value [c]	VIP [a]	fold-change [b]	P-Value [c]	VIP [a]	fold-change [b]	P-Value [c]	VIP [a]	fold-change [b]	P-Value [c]
4	1.090	1.953	0.004	0.936	1.664	0.007	1.295	2.267	0.000	1.068	5.438	0.000	1.230	3.870	0.000
6	1.272	0.285	0.000	0.467	0.684	0.122	0.369	1.086	0.757	0.507	1.098	0.566	0.438	1.162	0.757
11	1.152	1.850	0.005	0.813	1.258	0.047	1.246	1.701	0.001	1.115	3.706	0.000	1.101	1.909	0.001
13	1.161	1.806	0.002	1.305	2.482	0.000	1.334	2.975	0.000	1.089	3.327	0.000	1.211	5.009	0.000
15	1.038	1.851	0.012	1.118	1.876	0.004	1.000	1.859	0.024	1.004	3.090	0.001	1.193	4.583	0.000
20	1.151	1.813	0.004	1.139	1.550	0.007	1.225	1.714	0.001	1.070	2.894	0.000	1.229	2.413	0.000
21	1.190	1.902	0.002	1.093	1.417	0.007	1.098	1.525	0.007	1.111	5.566	0.000	1.188	2.915	0.000
26	1.206	2.066	0.001	1.283	2.070	0.000	1.214	1.961	0.001	1.073	2.995	0.000	1.098	1.816	0.001
27	1.154	0.441	0.003	1.136	0.432	0.003	0.691	1.009	0.965	1.036	4.223	0.001	0.936	1.780	0.004
28	1.129	0.626	0.009	1.304	0.430	0.000	0.674	0.955	0.627	1.013	2.052	0.001	0.704	1.116	0.453
31	1.176	1.826	0.002	1.009	1.354	0.038	1.241	2.166	0.001	1.118	5.199	0.000	1.203	2.913	0.000
32	1.091	1.864	0.002	1.072	1.694	0.004	0.882	1.616	0.015	1.098	5.304	0.000	1.041	2.513	0.001
33	1.040	0.686	0.024	0.761	0.794	0.085	0.740	1.071	0.825	1.056	3.487	0.000	0.810	1.434	0.171
35	1.054	1.557	0.009	0.688	0.875	0.233	1.010	1.505	0.024	1.084	2.770	0.000	0.820	1.133	0.354
36	1.051	0.712	0.012	0.921	0.772	0.024	1.219	0.577	0.001	0.514	0.973	0.627	1.173	0.492	0.000
37	1.432	0.083	0.000	1.430	0.092	0.000	1.409	0.090	0.000	1.109	0.217	0.000	1.281	0.141	0.000
38	1.279	0.326	0.000	1.323	0.216	0.000	1.331	0.221	0.000	0.807	0.542	0.007	1.254	0.180	0.000
39	1.279	1.995	0.001	1.292	1.809	0.001	1.332	2.360	0.000	1.128	5.221	0.000	1.268	3.469	0.000
43	1.376	0.135	0.000	1.411	0.097	0.000	1.313	0.122	0.000	0.929	0.506	0.003	1.260	0.108	0.000
44	1.268	3.941	0.001	1.302	6.369	0.000	1.262	5.444	0.000	1.083	6.830	0.000	1.250	9.829	0.000
45	1.240	1.879	0.001	1.182	1.779	0.003	1.228	1.839	0.000	1.100	3.305	0.000	1.122	1.850	0.001
55	1.285	3.218	0.000	1.390	3.771	0.000	1.289	4.135	0.000	1.036	5.254	0.000	1.170	3.596	0.000

56	1.287	4.312	0.000	1.383	8.653	0.000	1.385	5.741	0.000	1.064	5.361	0.000	1.205	5.609	0.000

[a] Calculated by orthogonal partial least-squares discriminant analysis (OPLS-DA) based on the relative contents of CLs in other groups compared with the HFD group.

[b] Changed multiple was calculated with relative contents of CLs in other groups compared to the HFD group.

[c] Calculated with SPSS software based on the relative contents of CLs in other groups compared with the HFD group.

Group information: ND, normal diet group; HFD, high-fat diet group; 0 DR, 20 DR, 40 DR and 60 DR refer to 0%, 20%, 40% and 60% dietary restriction relative to the normal diet group, respectively.
